# Patient-reported vision impairment in low luminance predicts multiple falls

**DOI:** 10.1186/s12877-023-04317-y

**Published:** 2023-09-21

**Authors:** Jan Henrik Terheyden, Johanna Gerhards, Reglind A. D. Ost, Maximilian W. M. Wintergerst, Frank G. Holz, Robert P. Finger

**Affiliations:** 1 Department of Ophthalmology, University Hospital Bonn, NRW, Venusberg-Campus 1, Ernst-Abbe-Str. 2, 53127 Bonn, Germany; 2 Department of Ophthalmology, University Hospital Mannheim & Medical Faculty Mannheim, University of Heidelberg, Mannheim, Germany

**Keywords:** Falling, Visual impairment, Patient-reported outcome, Risk factors

## Abstract

**Background:**

Visual impairment is an independent risk factor for falling. Whether this extends to patient-reported visual difficulties has not been assessed to date. We have evaluated whether patient-reported visual difficulties in low-contrast and low luminance situations are a risk factor for falls and concerns about falling.

**Methods:**

Baseline assessments in outpatients with varying degrees of visual impairment aged ≥ 60 years included the Vision Impairment in Low Luminance (VILL) questionnaire and socio-demographic data; prospective follow-up assessments included falls over 12 months, the Falls Efficacy Scale (FES-I) and the VILL. The VILL was scored using Rasch models, and the FES-I was categorized following published guidelines. Associations were investigated using logistic regression analysis, controlling for age, visual acuity and known risk factors of falling.

**Results:**

We included 112 participants (74 women, mean age 70 ± 7 years). Twenty-seven participants recalled any falls and eleven recalled multiple falls at follow-up. Higher VILL reading subscale and mobility subscale scores at baseline were significantly associated with reporting less multiple falls at follow-up (OR 0.559 [0.333–0.936], *p* = 0.027 and OR 0.595 [0.377–0.940], *p* = 0.026). VILL scores were significantly associated with concerns about falling (high versus low: *p* ≤ 0.004, reading, mobility and emotional subscales; high versus moderate: *p* = 0.004, emotional subscale).

**Conclusions:**

Patient-reported visual difficulties under low illumination and in low-contrast conditions are predictive of multiple falls in the future, have an additional predictive value over established risk scores, and are associated with concerns to fall. Current fall risk assessments may benefit from the inclusion of such assessments, e.g. the VILL questionnaire.

**Supplementary Information:**

The online version contains supplementary material available at 10.1186/s12877-023-04317-y.

## Background

Falls occur in more than 170 million people every year, resulting in almost 700,000 annual deaths and are important causes of morbidity and mortality in ageing populations worldwide [1]. Annual fall incidences in older adults further highlight the burden of fall events, ranging between 16 and 29% in Germany [2]. Reducing the number of falls may therefore contribute significantly to reducing the global disease burden in older adults. Visual impairment is a key risk factor for falls, more than doubling fall occurrences, and its prevalence is increasing globally [3, 4]. Besides visual function under daytime conditions, contrast sensitivity, and vision under low luminance, i.e. night-time conditions, have been reported as independent risk factors for falls [5–10].

Despite several international guidelines recommending an assessment of visual function in individuals at risk of falling [11–14], visual function testing is not widely performed when evaluating fall risk and is perceived as not feasible by a variety of healthcare providers [15, 16]. The prevalence of visual impairment increases considerably with age, affecting more than 1% of Germans above 60 years [17]. Patient-reports of perceived difficulties with tasks based on vision have been shown to correlate well with objective measures of visual function [18–20] and require little resources to be recorded. In line with this, various studies confirmed an association between self-reported vision and subsequent falling [8, 21–27]. While patient-report may provide a more feasible alternative to visual function tests in fall risk assessments, the available studies capture only vision under daylight conditions, whereas the value of assessing patient-reported vision under difficult lighting conditions including night-time has not been investigated in the context of falls prevention. The Vision Impairment in Low Luminance (VILL) questionnaire assesses patient-reported difficulties under low luminance and low contrast conditions and may therefore aid fall risk assessments [28–30].

Against this background, we assessed the association of patient-reported vision impairment in low luminance, visual function and subsequent falls as well as concerns about falling prospectively in a cohort of older adults.

## Methods

### Participants

Community-dwelling participants were recruited from the outpatient clinic of the Department of Ophthalmology, University Hospital Bonn, Germany between 2018 and 2022. All study procedures adhered to the tenets of the Declaration of Helsinki. All participants gave written informed consent, and the study protocol was approved by the institute’s committee on human research (ID 130/16). All individuals included in the study cohort were aged ≥ 60 years. We excluded cognitively impaired individuals (based on medical records) and illiterate individuals, and those with insufficient German language skills and/or reported acute-onset changes in vision / visual impairment.

### Study design

The study was a prospective observational proof-of-concept cohort study. Participants underwent two interviews which were conducted approximately one year apart. We initially administered the VILL questionnaire and a standardized questionnaire on socio-demographic characteristics (including age, sex, living situation, employment status) and medical history (ocular conditions, systemic diseases, to screen for exclusion criteria) as outlined previously [30]. The VILL questionnaire includes 33 items which focus on visual impairment and vision-related quality of life under challenging luminance and contrast conditions (Supplementary Table). To best reflect everyday conditions, participants are asked to consider their vision when wearing glasses or other visual aids, if applicable. The VILL consists of three subscales (reading and accessing information, mobility and safety, emotional well-being), and has been shown to be relevant to patients, content and construct valid, internally consistent, test–retest reliable, reliable across different modes of administration and associated with objectively measured visual function [28–30].

One year after the initial interview (minimum interval: 9 months), we conducted a second interview and asked participants to provide information on falls that had occurred over the previous 12 months. We assessed if and how many falls were remembered as well as additional fall characteristics (injuries secondary to falling, injuries requiring medical consultation, fractures secondary to falling). Falls were defined following the Prevention of Falls Network Europe (ProFaNE) definition [31]. Multiple falls were defined as ≥ two remembered fall events [32]. In addition, we recorded concerns about falling using the validated German version of the Falls Efficacy Scale International (FES-I), a 16-item instrument developed in the ProFaNE [33–35], as well as five established risk factors of falling beyond visual function (incontinence, cardiovascular diseases, vertigo, weakness of the lower limbs, depression) [36] and re-administered the VILL questionnaire. In summary, interview one included sociodemographic and medical history questions as well as the VILL questionnaire while interview two included the history of falling over the past 12 months, the FES-I and the VILL questionnaire.

### Questionnaire scoring

The VILL was scored using latent trait models, as explained previously [28–30]. In brief, we conducted Rasch analysis with Winsteps software (Chicago, IL) [37] to generate person measures for each subscale of the VILL, which minimizes the impact of missing responses and approximates interval-scaled scoring. The resulting scores can become positive or negative, with lower scores indicating a lower vision-related quality of life. The normality of the VILL score distributions was confirmed using quantile–quantile plots.

The scoring of the FES-I followed previously established recommendations [33–35]. In summary, a sum score with correction for missing responses was calculated. FES-I responses with more than four missing items were discarded. Concerns about falling were interpreted following previously established thresholds, i.e. low concern (total FES-I score 16–19 points), moderate concern (total score 20–27 points), or high concern (total score 28–64 points) [35].

### Statistical analysis

Demographic and questionnaire data were descriptively analysed. Results were compared between participants with and without a history of falling or multiple falls during the last year using the Mann Whitney U test and Fisher’s exact test. We calculated binary logistic regression models to investigate the association between VILL scores at baseline and reported falls or multiple falls at follow-up as primary outcomes, controlling for age, best-corrected visual acuity (VA) in the better eye (obtained from clinical records), and the number of the above-mentioned risk factors of falling. To examine associations between VILL scores and FES-I categories as secondary outcomes, we performed multinomial logistic regression analysis, also controlling for age, better eye VA, and the number of risk factors of falling (sum score with 1 point for each of the risk factors incontinence, cardiovascular diseases, vertigo, weakness of the lower limbs, depression). We used SPSS, version 27 (IBM Corporation, Armonk, New York) for the statistical analyses and considered *p*-values < 0.05 significant.

## Results

We included 112 participants (74 women, 66.1%) at a mean age of 70.1 ± 7.0 years (Table 1). Reasons for exclusion from an initial number of 127 participants were missed follow-up in thirteen individuals (reasons: Conflicting schedules, *n* = 11; death, *n* = 2) and an incomplete follow-up interview in 2 individuals. The follow-up interval was 584 ± 197 days on average and showed no significant association with reported falling (*p* = 0.434, Mann Whitney U test). Participants had 1.6 ± 1.2 risk factors of falling on average (Table 2) and varying degrees of visual impairment (VI), with 79.4% with no VI (VA ≤ 0.1 logarithm of the minimal angle of resolution [logMAR], i.e. ≥ 20/25), 18.5% with mild VI (0.2 logMAR ≤ VA ≤ 0.5 logMAR) and 2.1% with moderate or severe VI (VA ≥ 0.25 logMAR) based on best-corrected VA [38]. All participants suffered from ocular diseases, which included retinal diseases (59.8%), glaucoma (24.1%), cataract (30.4%), anterior segment conditions (9.8%) and other eye conditions (24.1%).

**Table 1:** Sample characteristics at baseline (*n* = 112)

		Mean ± SD or n(%)
Age [years]		70.1 ± 7.0
Sex	Male	38 (33.9%)
	Female	74 (66.1%)
Living situation	Alone	30 (26.8%)
	With others	81 (72.3%)
	Missing data	1 (0.9%)
Employment	Working	51 (45.5%)
	Unemployed	5 (4.5%)
	Retired	43 (38.4%)
	Missing data	13 (11.6%)
Better eye VA (LogMAR)		0.2 ± 0.2
VILL scores^a^	Reading subscale	0.3 ± 2.1
	Mobility subscale	0.1 ± 2.3
	Emotional subscale	2.0 ± 4.4

*FES-I* Falls Efficacy Scale, *VA* visual acuity, *LogMAR* logarithm of the minimum angle of resolution

**Fig. 1:**
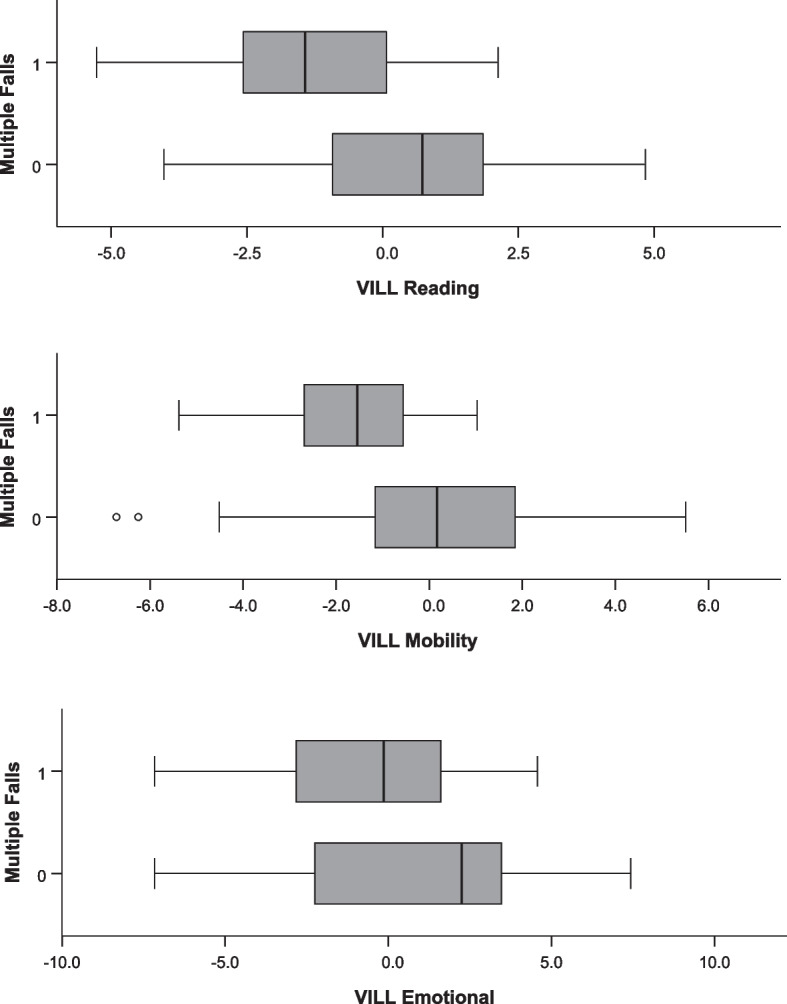
Boxplots of VILL person measures at baseline and reporting of multiple falls (1 – “yes” versus 0 – “no”) over 12 months. Higher VILL scores (obtained from Rasch models) indicate a better vision-related quality of life

**Fig. 2:**
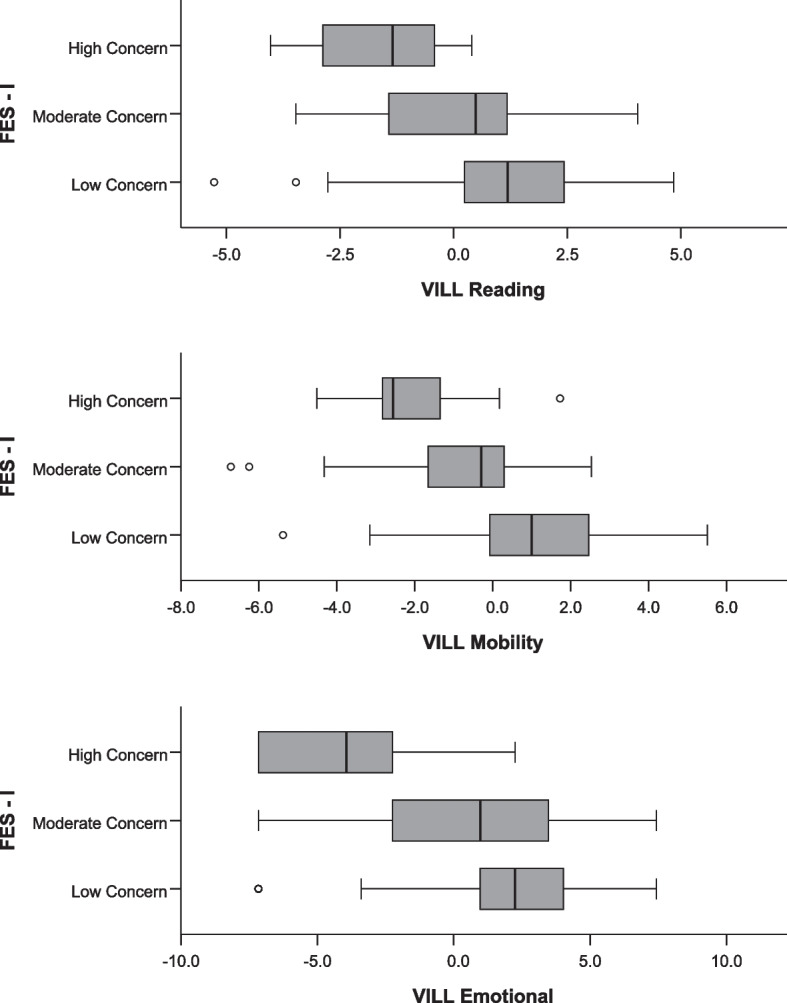
Boxplots of VILL person measures at baseline and concerns about falling interpretation categories from the FES-I

^a^VILL scores were generated based on Rasch models, with lower scores indicating a lower vision-related quality of life (distribution of scores, please see Figs. 1 and 2). Mean values are not directly comparable across subscales

**Table 2:** History of falls and concerns about falling reported at follow-up

		Mean ± SD or n(%)
Any Falls	Total	27 (24.1%)
	With injury	20 (17.9%)
	With injury + medical consultation	11 (9.8%)
Multiple falls	Total	11 (9.8%)
	With injury	9 (8.0%)
	With injury + medical consultation	5 (4.5%)
Risk factors	Total	1.6 ± 1.2
	Incontinence	40 (35.7%)
	Cardiovascular disease	65 (58.0%)
	Vertigo	40 (35.7%)
	Weakness of lower limbs	29 (25.9%)
	Depression	10 (8.9%)
FES-I	Sum score	22 ± 6
	Low concern	56 (50.0%)
	Moderate concern	40 (35.7%)
	High concern	16 (14.3%)

*FES-I* Falls Efficacy Scale, *VA* visual acuity, *LogMAR* logarithm of the minimum angle of resolution

Twenty-seven participants recalled falls in the last 12 months, of which 74% led to a medical consultation (Table 2). One participant reported a bone fracture secondary to falling (humerus fracture). The mean number of falls in the eleven participants who reported multiple falls was 2.8 ± 0.8 within a 12-month timeframe. Reported falling and multiple falling were not significantly associated with age, VA or any of the above-mentioned risk factors (*p* ≥ 0.323 and 0.116, respectively in Mann Whitney U test [age, VA] and Fisher’s exact test [risk factors]). Nonetheless, all analyses were corrected for these factors, since age, VA, and the additional risk factors may confound the association between patient-reported vision impairment in low luminance and reported falls. The mean number of reported falls in the groups that indicated low, moderate, and high concerns to fall were 1.6 ± 1.0 (13 participants), 1.6 ± 0.9 (12 participants) and 3.5 ± 0.7 (2 participants), respectively.

Higher self-reported reading and mobility scores (better vision-related quality of life) were significantly associated with a lower risk of multiple falls at follow-up in an adjusted binary logistic regression analysis (OR = 0.559 and 0.595, *p* = 0.027 and 0.026, respectively; Table 3; Fig. 1). When looking at multiple falls that led to injuries and consultations in the healthcare system, no significant associations with baseline VILL scores or changes in VILL scores over time were found (*p* ≥ 0.168 and 0.102 across VILL subscales, respectively). Neither of the VILL subscale scores at baseline were associated with any (minimum 1) falling event at follow-up when compared to no reported falling (*p* ≥ 0.071 across VILL subscales; Table 3). Worsening in individual VILL scores was not significantly associated with falling or multiple falling in our sample (*p* ≥ 0.236 across VILL subscales, adjusted for age, VA, risk factors of falling, and the follow-up time interval).

**Table 3:** Associations between VILL scores at baseline, reported falls and reported multiple falls at 12 months in binary logistic regression^a^. Higher VILL scores indicate better vision-related quality of life

Outcome	VILL subscale	Odds ratio	95% CI	*p*-value
Any Falls	Reading	0.759	0.562–1.024	0.071
	Mobility	0.816	0.628–1.062	0.130
	Emotional	1.083	0.942–1.246	0.262
Falls with injury	Reading	0.791	0.567–1.105	0.169
	Mobility	0.834	0.621–1.121	0.229
	Emotional	1.139	0.962–1.348	0.132
Multiple falls	Reading	0.559	0.333–0.936	**0.027**
	Mobility	0.595	0.377–0.940	**0.026**
	Emotional	0.921	0.756–1.123	0.418
Multiple falls with injury	Reading	0.732	0.441–1.215	0.228
	Mobility	0.725	0.460–1.145	0.168
	Emotional	0.996	0.797–1.246	0.975

*CI* confidence interval. *P*-values in bold were considered statistically significant

^a^adjusted for age, visual acuity and risk factors of falling

Higher scores of all VILL subscales were significantly associated with lower concerns to fall as measured by the FES-I (*p* ≤ 0.004, comparing “high concern” to “low concern” categories; Table 4; Fig. 2). Worsening VILL scores over time were not associated with concerns to fall (*p* ≥ 0.055 across VILL subscales) when adjusting for age, VA, risk factors of falling, and the follow-up interval.

**Table 4:** Associations between VILL scores at baseline and FES-I scores at 12 months in multinomial logistic regression (reference category: high concern)^a^. Higher VILL scores indicate better vision-related quality of life

Outcome	VILL subscale	FES-I Category	Odds ratio	95% CI	*p*-value
FES-I	Reading	Moderate concern	1.468	0.942–2.298	0.090
		Low concern	2.009	1.252–3.223	**0.004**
	Mobility	Moderate concern	1.212	0.816–1.800	0.341
		Low concern	2.001	1.283–3.120	**0.002**
	Emotional	Moderate concern	1.376	1.104–1.715	**0.004**
		Low concern	1.436	1.170–1.831	**0.001**

*CI* confidence interval, *FES-I* Falls Efficacy Scale. *P*-values in bold were considered statistically significant

^a^Adjusted for age, visual acuity and risk factors of falling

## Discussion

This study found that patient-reported visual impairment concerning reading and mobility tasks under low-luminance and low-contrast conditions using the VILL questionnaire could predict multiple falls in older adults over a 12-month period, when adjusting for age, VA and five established falling risk factors. This further highlights the need for assessing visual function in older adults for the prevention of falls and related complications. Assessments such as the VILL questionnaire are easily implemented in any setting, require very little previous staff training and provide additional valuable information relevant to falls and concerns about falling in the near future but pend further validation of our findings in a larger, geriatric sample.

Our results are well in line with previous studies, where self-reports of vision were significantly associated with falling [8, 21–27]. While these studies focused on self-assessments of vision under daylight conditions, our study specifically addresses visual difficulties under night-time conditions that occur with normal ageing and in many prevalent age-related eye diseases, such as age-related macular degeneration and glaucoma [39–41]. Yip and colleagues found a significant association between self-reported vision and falls in a population-based setting when controlling for VA (Odd’s ratio 1.28) [21]. We have also controlled our analyses for participants’ VA and our results support that patient-reported vision domains may be independent risk factors of subsequent falls compared to best-corrected VA alone. Due to the conceptual differences between the VILL and generic self-reports of vision, we are unable to comment on the relationship between these two patient-reports. Niihata et al. investigated visual functioning domains more specifically than other studies and identifed the domains of near vision, visual distress and role limitations due to vision to be significantly associated with falling but did not include any domains on night-time vision in a Japanese cohort [23]. The VILL subscale “reading and accessing information” partially corresponds to the near vision subscale assessed by Niihata et al., and both were significantly associated with falling. However, items from the VILL questionnaire have a focus on low-contrast and low-luminance reading (e.g. “Reading print which is not black”, “Recognizing small objects in dim lighting (e.g. coins)“) rather than vision under day-time conditions [29]. Interestingly, the “emotional well-being” subscale of the VILL questionnaire was not associated with falling in our study, which contrasts the results from the Japanese cohort.

The impact of falls on people’s lives can be devastating since falls are associated with an increased risk of fractures, head injury, depression, hospitalization and death [1, 3, 42, 43]. Thus, prevention of falls is increasingly important in our ageing societies and VI is one of the most relevant risk factors of falling, more than doubling the risk independently of other risk factors [3, 5, 7, 44, 45]. In a nationally representative survey conducted in the United States, more than 25% of visually impaired individuals reported multiple falls over the last 12 months [24]. Our data suggest that self-reported visual difficulties under low-luminance and low-contrast conditions using the VILL reading and mobility subscales may add to the assessment and prediction of risk of falling over and above objective measures of visual function.

Most studies investigating risk factors of falling have measured visual impairment based on VA under high illumination, i.e. daytime conditions. However, multiple population-based studies have reported contrast vision to be significantly associated with multiple falls [5–8]. A study in 156 community-dwelling older adults found contrast sensitivity and low-contrast visual acuity being even the strongest risk factors of multiple falling besides depth perception [9]. This is in accordance with our finding that a reduction in the reading and mobility subscales of the VILL questionnaire, which focuses on exactly these content domains, are significantly associated with multiple falls. However, our sample was recruited at an eye hospital and despite the majority of participants (79%) not being visually impaired, our approach requires to be further validated in an independent cohort. Interestingly, visual function parameters seem to be unrelated to any of these parameters when looking specifically at a low-vision population [46].

Implementing visual function tests in fall risk assessments is met with numerous challenges. This is particularly relevant for less common vision assessments such as contrast sensitivity or low luminance visual acuity, which become especially important in the context of fall risk assessments in older adults, as such assessments tend to be done by non-ophthalmic healthcare providers. Administration of patient-reported outcome measures (PROMs) on the contrary offers an easily implemented alternative which also yields valuable information related to visual functioning and visual difficulties adding to conventional fall risk assessments. Future studies should also assess the predictive value of assessing self-reported visual impairment in addition to patient-reported difficulties under low illumination.

In addition to reported falls, we also found patient-reported difficulties related to low-luminance and low-contrast vision using the VILL questionnaire to be associated with high concerns in the FES-I, a validated measure of fear of falling and self-efficacy [33–35]. Fear or falling itself is associated with activity restrictions, frailty, falling and institutionalization [47–49]. The significant association between the VILL and the FES-I in our dataset further supports the relevance of patient-reported visual difficulties in fall risk assessment. This is supported by the previous literature, where White et al. have identified significant associations between concerns to fall and contrast sensitivity measurements in a cohort of people with age-related macular degeneration [50, 51]. Interestingly, not only the functional (reading and mobility) subscales but also the emotional subscale of the VILL questionnaire were associated with the fear of falling, which may be explained by participants interpreting the concepts “concern” (as assessed by the FES-I) and “worry” (not assessed by the FES-I but related to emotional well-being) in similar ways.

Our study is the first to report an association of patient-reported visual difficulties under challenging light conditions with falls and concerns about falling. Strengths of our study include its prospective design, different outcome measures to support our findings, the comprehensive assessment of additional risk factors for falling and use of a variety of statistical techniques including Rasch models. Nevertheless, our study is limited by its relatively small sample size (11 participants reported multiple falls) and the heterogeneous population included which did not allow us to perform any subgroup analyses with the occurrence of falls as an outcome measure and limits the interpretability of the analyses of multiple falls with injuries, which were reported in less than ten participants. The inclusion criteria of our study were relatively wide and we cannot fully exclude selection bias based on this. Also, since participants were recruited at an eye hospital, selection bias may be present despite 79% of our study participants not being visually impaired. We adjusted our analyses for five recommended risk factors of falling but did not assess several other risk factors commonly associated with falls, including prior history of falling, polypharmacy, and cognitive impairment, or the availability of a caregiver, which may modify the associations. In addition, we had to rely on patient-reports of falls which is limited by memory bias and we did not make use of diaries, medical records or technical devices to objectify falls. We did not perform all follow-up interviews precisely 12 months after the first interview but expect this to have only limited impact on our results since VILL scores did not significantly change over time nor were reported falls associated with the length of the follow-up interval.

## Conclusions

Our results indicate that patient-reported visual difficulties under low-luminance and low-contrast conditions are predictive of multiple falls in the future and are associated with concerns to fall. Fall risk assessment may benefit from integrating this easily administered PROM independently of any objective visual function assessments. Future studies should assess how screening for patient-reported visual difficulties can be used to prevent multiple falls in the first place.

### Supplementary Information

**Additional file 1: Supplementary Table.** VILL items.

## Data Availability

The data that support the findings of this study are not publicly available as they contain information that could compromise participants’ privacy but are available from the corresponding author upon reasonable request.

## Abbreviations

FES-I: Falls Efficacy Scale
LogMAR: Logarithm of the minimal angle of resolution
PROM: Patient-reported outcome measure
VILL: Vision Impairment in Low Luminance questionnaire
