# Supplementary material for: Patient-reported vision impairment in low luminance predicts multiple falls
Source: BMC Geriatr. 2023 Sep 21;23:583. doi: 10.1186/s12877-023-04317-y (PMC10515075; doi:10.1186/s12877-023-04317-y)
Supplement: Supplementary file 1 — Additional file 1: Supplementary Table. VILL items. [file 12877_2023_4317_MOESM1_ESM.docx]

**Supplementary Table**: VILL items

| **Item** | **Subscale** |
| --- | --- |
| Adjusting to the dark when entering a dimly lit room (e.g. a restaurant at night) | Reading and accessing information |
| Recognizing small objects in dim lighting (e.g. coins) | Reading and accessing information |
| Recognizing people's faces outside during dusk | Reading and accessing information |
| Recognizing people or objects by candlelight | Reading and accessing information |
| Seeing things clearly close up in the middle of your field of vision | Reading and accessing information |
| Reading print which has a low contrast to its background | Reading and accessing information |
| Reading print which is not black (e.g. grey) | Reading and accessing information |
| Reading text on a digital display (e.g. in the car, on an electronic radio) | Reading and accessing information |
| Reading print against a colourful background (e.g. a brochure) | Reading and accessing information |
| Reading a paperback novel in dim lighting | Reading and accessing information |
| Reading a newspaper in dim lighting | Reading and accessing information |
| Reading a menu in a dimly lit restaurant | Reading and accessing information |
| Reading labels or instructions on medicine bottles in good lighting | Reading and accessing information |
| Reading labels or instructions on medicine bottles in dim lighting | Reading and accessing information |
| Reading package labels or price tags in a shop | Reading and accessing information |
| Driving a car on a sunny day (with or without sunglasses) | Mobility and safety |
| Driving a car along a road lined with trees on a sunny day (with or without sunglasses) | Mobility and safety |
| Driving a car at night | Mobility and safety |
| Driving a car at night in the rain | Mobility and safety |
| Reading street signs in time when driving by | Mobility and safety |
| Walking on uneven ground in the dark | Mobility and safety |
| Going out to do things during dusk (e.g. visiting the supermarket or shops) | Mobility and safety |
| Seeing steps or curbs in the dark | Mobility and safety |
| Getting your bearings in dimly lit or dark unfamiliar places | Mobility and safety |
| Felt blinded by oncoming cars at night | Mobility and safety |
| Felt blinded by the sun whilst driving a car (with or without sunglasses) | Mobility and safety |
| Felt unsafe as a pedestrian or cyclist at dawn or at night | Mobility and safety |
| Felt exhausted by reading in dim light | Reading and accessing information |
| Needed additional lighting to see or read anything | Reading and accessing information |
| Felt worried that your eyesight might get worse | Emotional well-being |
| Felt worried about losing your independence | Emotional well-being |
| Felt worried about the future | Emotional well-being |
| Felt worried that your lifestyle might change due to your eye condition | Emotional well-being |
